# Venereal Diseases in New York City

**Published:** 1913-07

**Authors:** 


"VENEREAL DISEASES IN NEW YORK CITY.—During the month of January, 1913, the Department of Health requested seven thousand physicians in Greater New York to furnish the department with information regarding the number of cases of syphilis, gonorrhea and chancroid that had been under their care in their private practice during the twelve months previous, in order that some idea of the number of cases of venereal disease treated during 1912 might be obtained. Only 2,215 physicians responded to this request, reporting 13,348 cases of syphilis, 24,980 cases of gonorrhea, and 4,431 cases of chancroid; making a total of 42,659 cases of venereal disease treated in the private practice of less than one-third of the physicians of this city within twelve months. The number of cases of syphilis is especially and alarmingly large. A complete census of the cases treated in institutions has been unobtainable up the present time, but the institutional cases, without doubt, exceed in number many times those treated in private practice. The apparently extensive prevalence of venereal diseases in New York City certainly furnishes food for thought."

I once saw a clown take a running start as if to leap over a high pole; instead of doing so, or attempting to do so, he *dodged under it.*

This simile comes to me on reading the above in the *New York Medical Journal* (June 21). After such a fearful showing (ven. dis. *epidemic* really, in New York) I thought the writer was going to *say something*. He says it. He says it is "food for thought."—"the apparently [sic] extensive prevalence."

Let me see: If 2,215 doctors report 42,659 cases, how many cases should 7000 doctors report? Stand up, Mary Jane, and answer. As 2,215 is to 7,000, so is 42,659 to the answer=139,990. This is in *private practice alone*, and does no include the hospitals, many times greater than in private practice.

The writer says the presence of an epidemic of venereal disease in New York is "food for thought." That's rather flat. It looks to me like food for very prompt and decisive action. Take the 139,990 cases for a basis and assume that there are as many more in hospitals, jails, institutions, etc., we can readily believe that it figures up to 300,000—a very large per cent of the entire population, and each one a focus of infection for the further spread. This insidious enemy is at work at the heart of our social system, strikes at the home, the family, and thus at the very integrity of the race! It renders marriage a hazardous proceeding even for the elect (?).

